# Correlates of Meeting the Physical Activity, Sedentary Behavior, and Sleep Guidelines for the Early Years among Belgian Preschool Children: The ToyBox-Study

**DOI:** 10.3390/ijerph17197006

**Published:** 2020-09-24

**Authors:** Marieke De Craemer, Vera Verbestel, Greet Cardon, Odysseas Androutsos, Yannis Manios, Sebastien Chastin

**Affiliations:** 1 Department of Rehabilitation Sciences, Ghent University, 9000 Ghent, Belgium; Vera.Verbestel@UGent.be; 2 Research Foundation Flanders, 1000 Brussels, Belgium; 3 Department of Movement and Sports Sciences, Ghent University, 9000 Ghent, Belgium; greet.cardon@UGent.be; 4 Department of Nutrition and Dietetics, School of Physical Education, Sport Science and Dietetics, University of Thessaly, 42100 Trikala, Greece; oandroutsos@uth.gr; 5 School of Health Sciences & Education, Department of Nutrition and Dietetics, Harokopio University, 17676 Athens, Greece; manios@hua.gr; 6 Department of Health and Community Sciences, Glasgow Caledonian University, Glasgow G4 0BA, UK; sebastien.chastin@gcu.ac.uk

**Keywords:** correlates, preschoolers, 24 h movement behaviors, physical activity, screen time, sleep duration

## Abstract

Physical activity, sedentary behavior, and sleep guidelines for preschool children were already established and integrated into the 24 h movement behavior guidelines in 2017. The aim of the current study was to investigate correlates of meeting or not meeting the physical activity, sedentary behavior, and sleep guidelines in Belgian preschool children. In total, 595 preschool children (53.3% boys, 46.7% girls, mean age: 4.2 years) provided complete data for the three behaviors and potentially associated correlates. Physical activity was objectively measured with accelerometers. Screen time, sleep duration, and correlates were reported by parents with the use of a questionnaire. Backward logistic regression was used to identify factors associated with meeting all guidelines for weekdays and weekend days. In the final model, older preschoolers (OR = 1.89), having a normal weight compared to being underweight (OR = 0.30), having parents that do not watch a lot of television (OR = 0.99), and having a father that attained higher education (OR = 1.91) were associated with meeting all guidelines on weekdays. For weekend days, a significant association was found for attending a sports club (OR = 1.08). Overall, only a few factors were associated with meeting the guidelines. A more comprehensive measurement of preschool children’s potential correlates of physical activity, sedentary behavior, and sleep is warranted.

## 1. Introduction

In the past, important lifestyle behaviors, such as physical activity, sedentary behavior, and sleep have mainly been studied in isolation [1,2,3,4]. Guidelines for physical activity [5], sedentary behavior [5], and sleep [6,7] were established for preschool children. The guidelines state that preschool children should be physically active for a minimum of 180 min per day at any intensity, to limit screen time to a maximum of 60 min per day, and to have good-quality sleep for 10 to 13 h each night. However, a recent shift in research emphasizes that these lifestyle behaviors are connected and are part of one 24 h day. If we look at the behaviors that everyone conducts within a 24 h timespan, every activity can be categorized as physical activity (categorized as light-intensity physical activity or moderate-to-vigorous physical activity), sedentary behavior, or sleep. The consequence of the co-dependence within one day is that a change in time spent on one or more behaviors affects the duration of at least one of the other behaviors [8]. In that context, 24 h movement behavior guidelines for preschoolers were launched by the World Health Organization (WHO) in May 2019, showing the importance and innovative aspect of this approach [9]. These 24 h movement behavior guidelines for the early years integrated and updated the pre-existing guidelines for physical activity, sedentary behavior, and sleep in this age group.

To our knowledge, only four studies have investigated compliance with all three guidelines, and all studies showed that less than 20% of preschoolers complied with the physical activity, sedentary behavior, and sleep guidelines, or the so-called 24 h movement behavior guidelines. These results were comparable across different countries, namely, Australia, Canada, Singapore, and Belgium [10,11,12,13]. These low compliance rates underline the need to develop and evaluate interventions that focus on the optimization of preschool children’s 24 h composition, i.e., optimal amounts of physical activity, sedentary behavior, and sleep across a 24 h timespan. The first step toward intervention development is investigating the correlates and determinants of these behaviors, as well as the composition of these behaviors. However, there are currently no studies that have investigated the associations between meeting or not meeting the physical activity, sedentary behavior, and sleep guidelines for the early years and potential correlates of meeting the guidelines.

Therefore, the aim of the current study was to investigate the potential correlates of meeting the physical activity, sedentary behavior, and sleep guidelines in a sample of Belgian preschool children in a manner that followed the socioecological model [14] (i.e., correlates at the individual, interpersonal, and organizational level). The choice of correlates was based upon the variables that were part of the assessment in a sample of Belgian preschool children participating in the European ToyBox-study. In addition, the literature shows the influence of correlates at the individual level (e.g., sex [15,16,17], socioeconomic status (SES) [18], age [18]), the interpersonal level (e.g., body mass index (BMI) of the parents [16,17], parental television (TV) time [17], screen time co-behavior [17], number of siblings in the house [17]), and the organizational level (e.g., afterschool childcare [19]) on one or more of these behaviors. Since previous studies additionally showed that behaviors might differ between weekdays and weekend days [20,21], potential correlates of meeting all three guidelines (i.e., physical activity, sedentary behavior, and sleep, also known as the 24 h movement behavior guidelines) were separately investigated for weekdays and weekend days.

## 2. Materials and Methods

### 2.1. Study Protocol

Participants in the present study were part of the baseline measurements of the European ToyBox-study (www.toybox-study.eu). A detailed description of the ToyBox-study was published elsewhere [4,22]. In brief, the aim of the ToyBox-study is to prevent preschoolers from becoming overweight or obese by developing, implementing, and evaluating a kindergarten-based, family-involved intervention in six European countries (Belgium, Bulgaria, Germany, Greece, Poland, and Spain). The Belgian baseline data were used in the current study since only in Belgium preschoolers’ physical activity levels were objectively measured using accelerometers. This study was approved by the Ethical Committee of the Ghent University Hospital (EC/2010/037).

For recruitment purposes, all cities and municipalities within the aforementioned provinces in each country were listed and ranked according to their SES (based on years of education for the population between 25 and 55 years old (cutoff: >14 years of education) or annual income at the level of the city or municipality (continuous variable) depending on the data available within the respective city or municipality). The list was then split into tertiles, resulting in a group of cities and municipalities with a low SES, a medium SES, and a high SES. From each tertile, five cities or municipalities were randomly selected (approximately five cities or municipalities for low SES, five for medium SES, and five for high SES). The random selection was conducted by the coordinating center (i.e., Harokopio University Athens, Greece). Preschools were then randomly selected from those municipalities (with the exclusion of the 20% of preschools with the smallest number of pupils). Ultimately, 27 Flemish preschools contributed to this study. All parents of the preschoolers (born in 2007 and 2008) received an information letter about the study and only children whose parents provided informed consent contributed to the study. Data collection occurred between May and June 2012 and consisted of preschoolers wearing an accelerometer and one of their parents filling in a questionnaire (Principal Caregiver’s Questionnaire). 

### 2.2. Measurements

#### 2.2.1. Physical Activity

How physical activity was measured has already been described elsewhere [10] and will therefore only briefly be described here. ActiGraph accelerometers (Pensacola, FL, USA; types GT1M, GT3X, and GT3X+) were used to objectively assess preschoolers’ physical activity. Since the GT1M accelerometer was validated to measure physical activity in preschoolers [23], and there is a strong agreement regarding the vertical axis counts among these types of accelerometers [24], these measurement devices were used in one study. The accelerometers were initialized to measure activity counts in 15 s epochs using ActiLife 5.5.5 software (Pensacola, FL, USA) [25]. Accelerometers were worn on the right hip and secured using an elastic waistband. The participating preschool children were asked to wear the accelerometer for six consecutive days (including two weekend days) during all waking hours. An information letter was handed out to preschool children’s parents with instructions on how to use the device to ensure correct handling of the device. After data collection, the data on the accelerometers were downloaded using ActifLife 5.5.5 software and reduced using Meterplus version 4.3 software (Santech Inc., San Diego, CA, USA). Data from both the first (i.e., fitting day, done by the researchers) and the sixth day (i.e., collection day) were omitted because the data from these days were incomplete. Nonwear time was defined as periods of ten minutes or more of consecutive zeros. To be included in the analyses, preschool children were required to have at least six hours of accelerometer recordings on a minimum of three days, including one weekend day [26]. The total physical activity time on weekdays and weekend days were categorized afterwards using the cutoff point of 275 counts/15 s, as proposed by Reilly et al. [27]. The total physical activity time was dichotomized into 0 (<180 min of total physical activity per day) and 1 (≥180 min of total physical activity per day) to calculate the proportion of preschoolers meeting the physical activity guideline of being physically active for more than 180 min per day, which was done separately for weekdays and weekend days. 

#### 2.2.2. Screen Time

Within the 24 h movement behavior guidelines, screen time is used as a proxy for total sedentary behavior. TV viewing and computer use were measured separately using two questions in the Principal Caregiver’s Questionnaire, which has been shown to be a reliable questionnaire [28]. More detail about the measurement of TV viewing and computer use has already been described elsewhere [10] and will therefore only briefly be described here. Both TV viewing and computer use were assessed separately for both weekdays and weekend days. For TV viewing, the question was formulated as follows: “About how many hours a day does your child usually watch TV (including DVDs and videos) in his/her free time?”. For computer use, the question was formulated as follows: “About how many hours a day does your child use the computer for activities like playing games on a computer, game consoles (e.g., Playstation, Xbox, GameCube) during leisure time?”. Answer possibilities were recoded into minutes of TV viewing and computer playing per day using the midpoint method [20] and were then added up to reflect the total screen time. The total screen time was dichotomized into 0 (>60 min of screen time per day) and 1 (≤60 min of screen time per day) to calculate the proportion of preschoolers meeting the screen time recommendation of less than one hour of screen time per day, separately for weekdays and weekend days.

#### 2.2.3. Sleep Duration

Sleep duration was assessed using one question in the Principal Caregiver’s Questionnaire. More detail about the measurement of sleep has already been described elsewhere [10] and will therefore only briefly be described here. The question was formulated separately for weekdays and weekend days as follows: “How many hours of sleep does your child usually have during the night?”. Answer possibilities stating “10–11 h” and “12–13 h” were coded as 1, reflecting the preschool children that met the sleep duration guidelines of 10 to 13 h of sleep per night. Answer possibilities stating sleep durations shorter or longer than 10 to 13 h of sleep per night were recoded as 0, reflecting all preschool children not meeting the sleep duration recommendations. To calculate the overall sleep duration, the answer possibilities were recoded into minutes of sleep by using the midpoint method [20], separately for weekdays and weekend days.

#### 2.2.4. Meeting All Guidelines

All preschool children who met all three guidelines (i.e., physical activity, TV viewing, and sleep) were coded as 1, and all preschool children who did not meet all three guidelines (i.e., also children meeting two out of three, and one out of three guidelines) were coded as 0, separately for weekdays and weekend days.

#### 2.2.5. Potential Correlates

The potential correlates were subdivided according to the three levels of the socioecological model and can be found below. The reason for including these potential correlates is that they were all investigated in the Principal Caregivers Questionnaire. Details on the correlates and how they were added to the analysis can be found in Table 1.

1. Individual-level correlates: age, sex, and weight status.
2. Interpersonal-level correlates: number of children in the household, parental BMI, parental age, parental TV viewing, parental computer time, parental SES, screen time co-behavior.
3. Organizational-level correlates: attending afterschool childcare, attending a sports club.

### 2.3. Statistical Analysis

Means and standard deviations for continuous variables and percentages for categorical variables were used to present the descriptive characteristics. The average time spent on total physical activity, screen time, and sleep was calculated separately for weekdays and weekend days. Furthermore, the proportion of preschool children meeting all three guidelines was calculated. Backward logistic regression analyses were conducted in MLwiN version 3.02 (University of Bristol, Bristol, UK). Multilevel modeling was used to take the clustering of preschool children within classes within preschools into account. Backward multilevel logistic regression analyses were conducted with preschoolers’ meeting all three guidelines as the dependent variable (separate for weekdays and weekend days), and the different possible correlates as the independent variables. First, multilevel regression analyses were performed for every category of correlates, including all correlates in those categories. For each step of the backward logistic regression, the correlate with the highest *p*-value was removed from the model. Eventually, the removal of correlates from the model was stopped once all correlates had a *p*-value below the threshold of 0.10. In total, two backward logistic regression analyses were performed, separately for weekdays and weekend days. The *p*-values, odds ratios (ORs), and 95% confidence intervals (95% CIs) are reported.

## 3. Results

### 3.1. Descriptive Characteristics of the Sample

An accelerometer was worn by 1082 preschool children, out of which 80.1% (*n* = 867) had a minimum of three days of valid data (including one weekend day) to make them eligible for the data analyses. Out of those 867 preschoolers, only 768 of them (72.6%) had complete data on the sleep behavior and screen time variables. Finally, 595 preschoolers of this sample were three and four years old (55.0%) and were included in the final sample since they had valid data for the outcome variables (i.e., sleep, physical activity, and sedentary behavior), and were thus included in the analyses. In Table 2, an overview of the descriptive characteristics is given. They were 4.20 years old (±0.46) on average, 53.3% were boys (*n* = 317), and 9.9% of the sample was overweight or obese (*n* = 59). The questionnaires were mostly filled out by the mother (*n* = 541, 90.9%), followed by 7.7% by the father (*n* = 46), both mother and father (*n* = 1, 0.2%), the stepmother (*n* = 1, 0.2%), and six parents (1.0%) did not specify who filled out the questionnaire.

In total, 9.9% of the preschool children (*n* = 59) met all three guidelines on weekdays, compared to 4.0% (*n* = 24) on weekend days. More detailed information on meeting guidelines of the three separate behaviors can be found in De Craemer et al. [10]. Preschool children were on average physically active for 145.50 (±39.46) min on weekdays, and 122.00 (±47.04) min on weekend days. They engaged in 65.03 (±49.70) min of screen time on weekdays and 119.24 (±82.45) min during weekend days. During weekdays and weekend days, preschool children slept for 10.96 (±1.07) h and 11.13 (±1.22) h per night, respectively.

### 3.2. Backward Logistic Regression Analysis

The backward logistic regression analysis for weekdays comprised 13 steps in which the correlate that was least significant was deleted from the model in every step. In the end, age (*p* = 0.09), weight status (with a significant association with underweight (*p* = 0.098)), parental TV viewing on weekdays (*p* = 0.04), and education of the father (*p* = 0.04) were retained in the final model (all *p* < 0.10).

The backward logistic regression analysis for weekend days comprised 16 steps. In the end, only attending a sports club was retained in the final model (*p* = 0.02).

### 3.3. Associations within the Final Model for Weekdays and Weekend Days

The results of the associations within the final model (separately for weekdays and weekend days) can be found in Table 3. In addition, the numbers of preschool children meeting or not meeting all three guidelines crossed with the categorical variables that were retained in the final model can be found in Supplementary Table S1.

Four correlates were left in the final model for weekdays, namely age, weight status, parental TV viewing on weekdays, and education of the father.

Older preschool children were 1.89 times more likely to meet the physical activity, sedentary behavior, and sleep guidelines on weekdays compared to younger children (95% CI = 1.16–2.62). Underweight preschool children were 3.33 times (1/0.30) less likely to meet all three guidelines compared with normal-weight children (95% CI = 1.15–1.74). Preschool children with parents engaging in higher levels of TV viewing during weekdays were 0.99 times less likely to meet all three guidelines (95% CI = 0.99–1.00), and preschool children with fathers having a higher education were 1.91 times more likely to meet all three guidelines (95% CI = 1.26–2.57). Only one correlate was retained in the final model for weekend days, namely attending a sports club. Preschool children attending a sports club were 1.08 times more likely to meet all three guidelines on weekend days compared to preschool children not attending a sports club (95% CI = 1.02–1.14).

## 4. Discussion

The aim of this study was to investigate the correlates for meeting physical activity, sedentary behavior, and sleep guidelines in preschool children, both for weekdays and weekend days. To answer this research question, data from the Belgian sample of the European ToyBox-study were used.

Few associations were found in the current study. For weekdays, four correlates were retained in the final model. These results show that older preschool children were more likely to meet the physical activity, sedentary behavior, and sleep guidelines on weekdays compared to younger preschool children. This underlines the fact that older preschool children are more likely to benefit from a structured school day compared to younger preschool children. A major influence for this is the kindergarten/preschool environment, in which structured physical education lessons and recess times are implemented, which induce higher physical activity levels during weekdays in comparison with weekend days, in which lower levels of physical activity are apparent in preschool children [34]. In addition, it might be possible that preschool children watch less TV and that they more often go to sleep at set times during schooldays/weekdays compared to weekend days. Another recent study showed similar results with older preschool children engaging in higher levels of moderate-to-vigorous physical activity and less sedentary behavior compared to younger preschool children [35]. Together with the study by Nilsen et al., results from the current study suggest that the kindergarten/preschool environment appears to be important for developing physical activity and movement behaviors [35]. Weight status was also retained in the final model, which showed that normal-weight preschool children were more likely to meet the guidelines compared to underweight children. Furthermore, preschool children having parents that watch a lot of TV on weekdays were less likely to meet the guidelines. This result underlines previous results from the literature showing that parents are important role models in the life of their children and that parental behavior has an influence on children’s behavior [15,16,17]. A recent study in Chinese primary school children also showed the mediating role of children’s screen-viewing time in the association between parental screen-viewing and children being overweight. A study by Lin et al. showed that parents decreasing their screen time effectively decreases children’s screen-viewing time, which has an influence on children’s weight status [36]. In addition, besides the preschool environment, the home environment is a setting where preschool children spend most of their time [37]. This underlines the fact that parents have an important influence and actively involving parents in the development of future interventions might be a good strategy.

Finally, father’s education was also retained in the final model and this result showed that preschool children having a father that attained higher education were more likely to meet the guidelines. Therefore, it is important to focus on preschool children from lower SES when looking at the combined physical activity, sedentary behavior, and sleep guidelines. On weekend days, only attending a sports club appeared to be significantly associated with meeting all three guidelines, showing that membership of a sports club was possibly confounded with meeting the guidelines at a young age.

Currently, this is the first study to our knowledge that investigates correlates that are associated with meeting or not meeting all three guidelines (i.e., physical activity, sedentary behavior, and sleep) in preschool children. Other studies already investigated meeting the newly established 24 h movement behavior guidelines and indicators of adiposity [12,38], but this study is the first to investigate possible correlates, such as correlates at the individual, interpersonal, and organizational levels. One of the possible reasons for finding only limited associations might be the fact that preschool children are still young and it might be possible that more associations between correlates and meeting the physical activity, sedentary behavior, and sleep guidelines for youth will appear once preschool children get older and belong to a different age group [39]. Although this study was cross-sectional, it has a high value in this age group due to the relatively small period that preschool children can be followed up. However, given that this was a cross-sectional study with multiple comparisons and small effects, it was impossible to draw causal conclusions. Therefore, longitudinal studies might provide us with better insight into whether compliance with 24 h movement behavior guidelines tracks into later life. Therefore, future studies should investigate preschoolers’ compliance with all three guidelines, also known as the 24 h movement behavior guidelines, by using a prospective design in which several measurements of the 24 h movement behaviors are conducted and consequently compared with the 24 h movement behavior guidelines for other age groups. In addition, this study only considered a small and limited number of potential correlates, which were mostly individual-level variables. However, the environment, including the home and kindergarten environment, might be important too. Therefore, future research needs to consider a more comprehensive set of potential correlates and their interactions [40,41].

The current study included a large amount of data on Belgian preschool children’s physical activity, sedentary behavior, and sleep, which is a strength of the current study. In addition, accelerometers were used to objectively measure preschool children’s physical activity. A limitation of the current study is the fact that a questionnaire was used to measure screen time, where parents had to report on their children’s behavior. This might have induced a social desirability bias, and it is difficult for parents to accurately report on their child’s behavior. Future studies should objectively measure preschool children’s physical activity, sedentary behavior, and sleep with the use of, for example, an ActivPAL accelerometer, which includes an inclinometer that can distinguish between sitting (i.e., sedentary behavior) and standing (i.e., light physical activity). Furthermore, the questionnaire used in the current study was not drafted for this particular aim, which means that the screen time and sleep duration questions might be optimized in future studies using proxy-reported questionnaires. In addition, only a limited amount of correlates could be analyzed due to the fact that this was not the aim of the original study. Within the ToyBox-study, focus groups and systematic reviews were conducted to identify correlates of physical activity and sedentary behavior in preschool children, but there was no focus on sleep and correlates of sleep in preschool children. Therefore, researchers of future studies investigating the correlates of preschool children’s movement behaviors should carefully think about possible influencing factors/correlates/determinants of preschoolers’ movement behaviors before conducting their studies. Additionally, the dataset used in the current study was collected in 2012, which means that the included data are relatively old and the access to screen devices, such as smartphones and tablets, has changed since the data collection. For that reason, the understanding of our results might not be applicable to these new and mobile screen devices. Data collection regarding the use of mobile screen devices (i.e., smartphones, tablets) in combination with the amount of television and computer time should be included in future studies. Finally, it is important to mention that only a small proportion of preschool children met the guidelines on weekdays (9.9%) and weekend days (4.0%), which means that preschool children who met the guidelines and preschool children who did not meet the guidelines were unequally distributed, and the sample of preschoolers meeting the guidelines was limited as a result. Thus, the results should be interpreted with caution. Future studies should recruit larger samples to overcome this issue.

## 5. Conclusions

Overall, few correlates of meeting the guidelines were found. A more comprehensive set of potential preschool children’s correlates of physical activity, sedentary behavior, and sleep should be considered in future studies. In addition, this research could also be expanded to more regions or countries.

## Figures and Tables

**Table 1:** Details of the correlates *.

Correlate	How It Was Measured in the Questionnaire	How It Was Added to the Analyses
**Individual-Level Correlates**		
Age	Preschoolers’ date of birth, as reported by the parents.	Age at date of data collection. Ratio scale variable.
Sex	What is the sex of your child? (1 = boy, 2 = girl).	Girl = 0 = reference category Boy = 1
Weight status	Preschoolers’ height and weight were measured at the preschools by trained research assistants according to standardized protocols [29]. Children were measured in light clothing without shoes. Height (inter-observer reliability: 98.1%) was measured with a SECA 225 Leicester Portable stadiometer (accuracy of 0.1 cm) (SECA, Hamburg, Germany). Weight (inter-observer reliability: 99.9%) was measured with a calibrated electronic scale SECA 861 (accuracy of 0.1 kg) (SECA, Hamburg, Germany). For each measurement, two readings were obtained and the mean was used for the analyses. When the two readings differed by more than 1%, a third measurement was conducted and the mean of the two least differing values was used.	BMI was calculated as weight/height^2^ (kg/m^2^). Weight status (underweight, normal weight, overweight, obese) was obtained according to the International Obesity Task Force thresholds [30]. Four categories with three codes: Underweight = 0 = reference category Normal weight = 1 Overweight or obese = 2
Waist circumference	Preschoolers’ waist circumference was measured at the preschools by trained research assistants according to standardized protocols [29]. Children were measured in light clothing without shoes. Waist circumference was measured with a SECA 200 or SECA 201 (SECA, Hamburg, Germany) to the nearest 0.1 cm. For each measurement, two readings were obtained and the mean was used for the analyses. When the two readings differed by more than 1%, a third measurement was conducted and the mean of the two least differing values was used.	Ratio scale variable
**Interpersonal-Level Correlates**		
Number of children in the household	How many children below the age of 18 years are living permanently in the household? (open question).	Ratio scale variable.
Parental BMI	Self-reported height and weight, both for fathers and mothers.	BMI was calculated (kg/m^2^). Ratio scale variable. Separately for fathers and mothers.
Parental age	Self-reported, both for fathers and mothers.	Ratio scale variable. Separately for fathers and mothers.
Parental TV viewing	For weekdays and weekend days: About how many hours a day do you watch TV (including DVDs and videos) in your free time? (Answer possibilities: never, less than 30 min/day, 30 min to <1 h/day, 1–2 h/day, 3–4 h/day, 5–6 h/day, 7–8 h/day, 8 h per day, more than 8 h/day, I don’t know).	Answer possibilities were recoded into minutes of TV viewing per day using the midpoint method [20]. Ratio scale variable.
Parental computer time	For weekdays and weekend days: About how many hours a day do you usually use the computer for activities like chatting online, internet, emailing, playing games, and/or do you play game consoles (e.g., Playstation, Xbox, GameCube) during leisure time? (Answer possibilities: never, less than 30 min/day, 30 min to <1 h/day, 1–2 h/day, 3–4 h/day, 5–6 h/day, 7–8 h/day, 8 h per day, more than 8 h/day, I don’t know).	Answer possibilities were recoded into minutes of computer time per day using the midpoint method [20]. Ratio scale variable.
Parental SES	Parental education was used as an SES indicator [31]. Starting from the age of six, how many years of education did you complete? (Answer possibilities: less than 7 years, 7–12 years, 13–14 years, 15–16 years, more than 16 years). Separately for mothers and fathers.	Fathers’ and mothers’ educational level was dichotomized into lower SES and higher SES. This distinguished parents who have completed medium or higher education, college, or university from other families [32]. 14 years of education or less = 0 = reference category More than 14 years of education = 1
Screen time co-behavior (i.e., watching TV, DVD, or videos together with the child)	How often do you or your spouse/partner watch TV, DVD/video together with your child? (Answer possibilities: never, less than once a week, once a week, 2–4 days/week, 5–6 days/week, every day (once a day), every day (more than once a day)).	Six categories: Never = 0 = reference category Less than once a week = 1 Once a week = 2 2–4 days/week = 3 5–6 days/week = 4 Every day (once or more than once a day) = 5
**Organizational-Level Correlates**		
Attending after-school childcare	Two questions were used to calculate the hours of after-school childcare per week. How many days per week does your child usually attend childcare? (open question). How many hours per day does your child usually attend childcare? (open question).	Days were multiplied with the hours. Ratio scale variable.
Attending a sports club	Does your child attend a sports club? (yes = 1, no = 2).	No = 0 Yes = 1

* Detailed information regarding the procedures and training of research staff and the validity and reliability of the measurements were published elsewhere [33]. BMI—body mass index, SES—socioeconomic status.

**Table 2:** Descriptive characteristics of the total sample (*n* = 595).

Variables	Means and Standard Deviations/Percentages
**Physical Activity, Screen Time, and Sleep**	
Total physical activity (min/day)	
Weekday	145.50 ± 39.46
Weekend day	122.00 ± 47.04
Screen time (min/day)	
Weekday	65.03 ± 49.70
Weekend day	119.24 ± 82.45
Sleep duration (h/day)	
Weekday	10.96 ± 1.07
Weekend day	11.13 ± 1.22
Meeting/not meeting all three guidelines weekdays (%)	
Meeting	9.9 (*n* = 59)
Not meeting	90.1 (*n* = 536)
Meeting/not meeting all three guidelines weekend days (%)	
Meeting	4.0 (*n* = 24)
Not meeting	96.0 (*n* = 571)
**Individual-Level Correlates**	
Age (years)	4.20 ± 0.46
Sex (% boys)	53.3
BMI (kg/m^2^)	15.87 ± 1.29
Weight status (%)	
Underweight	10.5
Normal weight	79
Overweight or obese	10.5
Waist circumference (cm)	51.38 ± 3.28
**Interpersonal-Level Correlates**	
Number of children in the household	2.03 ± 0.88
BMI mother (kg/m^2^)	23.54 ± 3.91
BMI father (kg/m^2^)	25.24 ± 3.40
Age mother (years)	33.48 ± 4.26
Age father (years)	36.05 ± 5.14
Parental TV viewing weekdays (min/day)	94.40 ± 65.13
Parental TV viewing weekend days (min/day)	130.36 ± 80.56
Parental computer time weekdays (min/day)	50.54 ± 74.57
Parental computer time weekend days (min/day)	50.37 ± 62.11
Mother’s educational level (%)	
Lower education (14 years or less)	33
Higher education (more than 14 years)	67
Father’s educational level (%)	
Lower education (14 years or less)	45.7
Higher education (more than 14 years)	54.3
Screen time co-behavior with the child (%)	
Never	4.4
Less than once a week	24.6
Once a week	24.3
2–4 days a week	22.1
5–6 days a week	5.8
Every day, once or more than once a day	18.8
**Organizational-Level Correlates**	
Attending after-school childcare (h/week)	3.23 ± 4.48
Attending a sports club (%)	
Yes	31.9
No	68.1

**Table 3:** Results of the final multilevel logistic regression analyses, with meeting physical activity, sedentary behavior, and sleep guidelines as the dependent variables, separately for weekdays and weekend days.

Correlate	*β* ± Standard Error	Odds Ratio	95% Confidence Interval
**Weekdays (*χ*^2^ = 14.84, *p* = 0.01)**			
Age	0.64 ± 0.37	1.89	1.16–2.62
Weight status (ref = normal weight) Underweight	−1.22 ± 0.74	0.30	1.15–1.74
Parental TV viewing weekdays	−0.006 ± 0.003	0.99	0.99–1.00
Education father	0.65 ± 0.33	1.91	1.26–2.57
**Weekend Days (*χ*^2^ = 5.60, *p* = 0.02)**			
Attending a sports club	0.08 ± 0.03	1.08	1.02–1.14

## Supplementary Materials

The following are available online at https://www.mdpi.com/1660-4601/17/19/7006/s1, Table S1: Numbers of preschool children meeting or not meeting all three guidelines crossed with the categorical variables that were retained in the final model.

## References

[1] O’Dwyer M.V., Fairclough S.J., Ridgers N.D., Knowles Z.R., Foweather L., Stratton G. (2013). Effect of a school-based active play intervention on sedentary time and physical activity in preschool children. Health Educ. Res..

[2] Reilly J.J., Kelly L., Montgomery C., Williamson A., Fisher A., McColl J.H., Lo Conte R., Paton J.Y., Grant S. (2006). Physical activity to prevent obesity in young children: Cluster randomised controlled trial. Br. Med. J..

[3] Pedisic Z. (2014). Measurement Issues and Poor Adjustments for Physical Activity and Sleep Undermine Sedentary Behaviour Research—The Focus Should Shift to the Balance between Sleep, Sedentary Behaviour, Standing and Activity. Kinesiology.

[4] Manios Y., Androutsos O., Katsarou C., Iotova V., Socha P., Geyer C., Moreno L., Koletzko B., De Bourdeaudhuij I. (2014). Designing and implementing a kindergarten-based, family-involved intervention to prevent obesity in early childhood: The ToyBox-study. Obes. Rev..

[5] Australian Government—Department of Health Move and Play Every Day—National Physical Activity Recommendations for Children 0–5 Years. https://www1.health.gov.au/internet/main/publishing.nsf/Content/F01F92328EDADA5BCA257BF0001E720D/$File/FS%200-5yrs.PDF.

[6] Paruthi S., Brooks L.J., D’Ambrosio C., Hall W.A., Kotagal S., Lloyd R.M., Malow B.A., Maski K., Nichols C., Quan S.F. (2016). Consensus statement of the american academy of sleep medicine on the recommended amount of sleep for healthy children: Methodology and discussion. J. Clin. Sleep Med..

[7] Hirshkowitz M., Whiton K., Albert S.M., Alessi C., Bruni O., DonCarlos L., Hazen N., Herman J., Adams Hillard P.J., Katz E.S. (2015). National sleep foundation’s updated sleep duration recommendations: Final report. Sleep Health.

[8] Chaput J.P., Carson V., Gray C.E., Tremblay M.S. (2014). Importance of all movement behaviors in a 24 h period for overall health. Int. J. Environ. Res. Public Health.

[9] World Health Organization Guidelines on Physical Activity, Sedentary Behaviour and Sleep for Children under 5 Years of Age. https://apps.who.int/iris/handle/10665/311664.

[10] De Craemer M., McGregor D., Androutsos O., Manios Y., Cardon G. (2018). Compliance with 24-h Movement Behaviour Guidelines among Belgian Pre-School Children: The ToyBox-Study. Int. J. Environ. Res. Public Health.

[11] Chen B., Bernard J.Y., Padmapriya N., Yao J., Goh C., Tan K.H., Yap F., Chong Y.S., Shek L., Godfrey K.M. (2019). Socio-demographic and maternal predictors of adherence to 24-h movement guidelines in Singaporean children. Int. J. Behav. Nutr. Phys. Act..

[12] Chaput J.P., Colley R.C., Aubert S., Carson V., Janssen I., Roberts K.C., Tremblay M.S. (2017). Proportion of preschool-aged children meeting the Canadian 24-Hour Movement Guidelines and associations with adiposity: Results from the Canadian Health Measures Survey. BMC Public Health.

[13] Cliff D.P., McNeill J., Vella S.A., Howard S.J., Santos R., Batterham M., Melhuish E., Okely A.D., de Rosnay M. (2017). Adherence to 24-h movement guidelines for the early years and associations with social-cognitive development among Australian preschool children. BMC Public Health.

[14] Sallis J.F., Owen N., Fisher E.B., Glanz K., Rimer B.K., Viswanath K. (2008). Ecological models of health behavior. Health Behavior and Health Education: Theory, Research, and Practice.

[15] Hinkley T., Crawford D., Salmon J., Okely A.D., Hesketh K. (2008). Preschool children and physical activity: A review of correlates. Am. J. Prev. Med..

[16] Timmons B.W., Naylor P.J., Pfeiffer K.A. (2007). Physical activity for preschool children—How much and how?. Can. J. Public Health.

[17] De Craemer M., De Decker E., De Bourdeaudhuij I., Vereecken C., Deforche B., Manios Y., Cardon G., ToyBox-Study Group (2012). Correlates of energy balance-related behaviours in preschool children: A systematic review. Obes. Rev..

[18] Hoyos Cillero I., Jago R. (2010). Systematic review of correlates of screen-viewing among young children. Prev. Med..

[19] Trost S.G., Rosenkranz R.R., Dzewaltowski D. (2008). Physical activity levels among children attending after-school programs. Med. Sci. Sports Exerc..

[20] De Craemer M., Lateva M., Iotova V., De Decker E., Verloigne M., De Bourdeaudhuij I., Androutsos O., Socha P., Kulaga Z., Moreno L. (2015). Differences in energy balance-related behaviours in European preschool children: The ToyBox-study. PLoS ONE.

[21] Van Stappen V., Van Dyck D., Latomme J., De Bourdeaudhuij I., Moreno L., Socha P., Iotova V., Koletzko B., Manios Y., Androutsos O. (2018). Daily patterns of preschoolers’ objectively measured step counts in six european countries: Cross-sectional results from the ToyBox-Study. Int. J. Environ. Res. Public Health.

[22] Manios Y., Grammatikaki E., Androutsos O., Chinapaw M.J., Gibson E.L., Buijs G., Iotova V., Socha P., Annemans L., Wildgruber A. (2012). A systematic approach for the development of a kindergarten-based intervention for the prevention of obesity in preschool age children: The ToyBox-study. Obes. Rev..

[23] Pate R.R., O’Neill J.R., Mitchell J. (2010). Measurement of physical activity in preschool children. Med. Sci. Sports Exerc..

[24] Robusto K.M., Trost S.G. (2012). Comparison of three generations of ActiGraph activity monitors in children and adolescents. J. Sports Sci..

[25] Nilsson A., Ekelund U., Yngve A., Sjostrom M. (2002). Assessing physical activity among children with accelerometers using different time sampling intervals and placements. Pediatr. Exerc. Sci..

[26] Penpraze V., Reilly J.J., MacLean C.M., Montgomery C., Kelly L.A., Paton J.Y., Aitchison T., Grant S. (2006). Monitoring of physical activity in young children: How much is enough?. Pediatr. Exerc. Sci..

[27] Reilly J.J., Coyle J., Kelly L., Burke G., Grant S., Paton J.Y. (2003). An objective method for measurement of sedentary behavior in 3- to 4-year olds. Obes. Res..

[28] Gonzalez-Gil E.M., Mouratidou T., Cardon G., Androutsos O., De Bourdeaudhuij I., Gozdz M., Usheva N., Birnbaum J., Manios Y., Moreno L.A. (2014). Reliability of primary caregivers reports on lifestyle behaviours of European pre-school children: The ToyBox-study. Obes. Rev..

[29] Mouratidou T., Miguel M.L., Androutsos O., Manios Y., De Bourdeaudhuij I., Cardon G., Kulaga Z., Socha P., Galcheva S., Iotova V. (2014). Tools, harmonization and standardization procedures of the impact and outcome evaluation indices obtained during a kindergarten-based, family-involved intervention to prevent obesity in early childhood: The ToyBox-study. Obes. Rev..

[30] Cole T.J., Lobstein T. (2012). Extended international (IOTF) body mass index cut-offs for thinness, overweight and obesity. Pediatric Obes..

[31] Winkleby M.A., Jatulis D.E., Frank E., Fortmann S.P. (1992). Socioeconomic status and health: How education, income, and occupation contribute to risk factors for cardiovascular disease. Am. J. Public Health.

[32] Brug J., van Stralen M.M., Te Velde S.J., Chinapaw M.J., De Bourdeaudhuij I., Lien N., Bere E., Maskini V., Singh A.S., Maes L. (2012). Differences in weight status and energy-balance related behaviors among schoolchildren across Europe: The ENERGY-project. PLoS ONE.

[33] De Miguel-Etayo P., Mesana M.I., Cardon G., De Bourdeaudhuij I., Gozdz M., Socha P., Lateva M., Iotova V., Koletzko B.V., Duvinage K. (2014). Reliability of anthropometric measurements in European preschool children: The ToyBox-study. Obes. Rev..

[34] Verbestel V., Van Cauwenberghe E., De Coen V., Maes L., De Bourdeaudhuij I., Cardon G. (2011). Within- and between-day variability of objectively measured physical activity in preschoolers. Pediatric Exerc. Sci..

[35] Nilsen A.K.O., Anderssen S.A., Resaland G.K., Johannessen K., Ylvisaaker E., Aadland E. (2019). Boys, older children, and highly active children benefit most from the preschool arena regarding moderate-to-vigorous physical activity: A cross-sectional study of Norwegian preschoolers. Prev. Med. Rep..

[36] Lin Y.C., Tsai M.C., Strong C., Hsieh Y.P., Lin C.Y., Lee C.S.C. (2020). Exploring mediation roles of child screen-viewing between parental factors and child overweight in Taiwan. Int. J. Environ. Res. Public Health.

[37] Golan M. (2006). Parents as agents of change in childhood obesity--from research to practice. Int. J. Pediatric Obes..

[38] Santos R., Zhang Z., Pereira J.R., Sousa-Sa E., Cliff D.P., Okely A.D. (2017). Compliance with the Australian 24-h movement guidelines for the early years: Associations with weight status. BMC Public Health.

[39] Lee E.Y., Carson V., Jeon J.Y., Spence J.C., Tremblay M.S. (2019). Levels and correlates of 24-h movement behaviors among South Koreans: Results from the Korea National Health and Nutrition Examination Surveys, 2014 and 2015. J. Sport Health Sci..

[40] Condello G., Ling F.C., Bianco A., Chastin S., Cardon G., Ciarapica D., Conte D., Cortis C., De Craemer M., Di Blasio A. (2016). Using concept mapping in the development of the EU-PAD framework (EUropean-Physical Activity Determinants across the life course): A DEDIPAC-study. BMC Public Health.

[41] Chastin S.F., De Craemer M., Lien N., Bernaards C., Buck C., Oppert J.M., Nazare J.A., Lakerveld J., O’Donoghue G., Holdsworth M. (2016). The SOS-framework (Systems of Sedentary behaviours): An international transdisciplinary consensus framework for the study of determinants, research priorities and policy on sedentary behaviour across the life course: A DEDIPAC-study. Int. J. Behav. Nutr. Phys. Act..

