# Supplementary material for: Correlates of Meeting the Physical Activity, Sedentary Behavior, and Sleep Guidelines for the Early Years among Belgian Preschool Children: The ToyBox-Study
Source: Int J Environ Res Public Health. 2020 Sep 24;17(19):7006. doi: 10.3390/ijerph17197006 (PMC7579535; doi:10.3390/ijerph17197006)
Supplement: Supplementary file 1 [file ijerph-17-07006-s001.pdf]

**Supplementary Table 1.** Numbers of preschool children meeting or not meeting all three guidelines crossed with the categorical variables that were retained in the final model.			
		Meeting all three guidelines	Not meeting all three guidelines
Weight status	Underweight	3	56
	Normal weight	48	397
	Overweight/obese	4	55
Education father	Low SES	18	235
	High SES	38	263
Attending a sports club	No	13	386
	Yes	11	176
